# Age‐related structural and functional variations in 5,967 individuals across the adult lifespan

**DOI:** 10.1002/hbm.24905

**Published:** 2019-12-26

**Authors:** Na Luo, Jing Sui, Anees Abrol, Dongdong Lin, Jiayu Chen, Victor M. Vergara, Zening Fu, Yuhui Du, Eswar Damaraju, Yong Xu, Jessica A. Turner, Vince D. Calhoun

**Affiliations:** ^1^ Brainnetome Center and National Laboratory of Pattern Recognition Institute of Automation, Chinese Academy of Sciences Beijing China; ^2^ University of Chinese Academy of Sciences Beijing China; ^3^ CAS Center for Excellence in Brain Science and Intelligence Technology Institute of Automation, Chinese Academy of Sciences Beijing China; ^4^ Tri‐Institutional Center for Translational Research in Neuroimaging and Data Science (TReNDS): Georgia State University, Georgia Institute of Technology, and Emory University Atlanta Georgia; ^5^ School of Computer and Information Technology Shanxi University Taiyuan China; ^6^ Department of Psychiatry First Clinical Medical College/ First Hospital of Shanxi Medical University Taiyuan China; ^7^ Department of Psychology Neuroscience Institute, Georgia State University Atlanta Georgia; ^8^ Department of Psychiatry Yale University, School of Medicine New Haven Connecticut; ^9^ Department of Psychology, Computer Science Neuroscience Institute, and Physics, Georgia State University Atlanta Georgia; ^10^ Department of Electrical and Computer Engineering Georgia Institute of Technology Atlanta Georgia

**Keywords:** adult lifespan, age‐related variations, functional network connectivity, independent component analysis, multivariate linear regression model, structural network correlation

## Abstract

Exploring brain changes across the human lifespan is becoming an important topic in neuroscience. Though there are multiple studies which investigated the relationship between age and brain imaging, the results are heterogeneous due to small sample sizes and relatively narrow age ranges. Here, based on year‐wise estimation of 5,967 subjects from 13 to 72 years old, we aimed to provide a more precise description of adult lifespan variation trajectories of gray matter volume (GMV), structural network correlation (SNC), and functional network connectivity (FNC) using independent component analysis and multivariate linear regression model. Our results revealed the following relationships: (a) GMV linearly declined with age in most regions, while parahippocampus showed an inverted U‐shape quadratic relationship with age; SNC presented a U‐shape quadratic relationship with age within cerebellum, and inverted U‐shape relationship primarily in the default mode network (DMN) and frontoparietal (FP) related correlation. (b) FNC tended to linearly decrease within resting‐state networks (RSNs), especially in the visual network and DMN. Early increase was revealed between RSNs, primarily in FP and DMN, which experienced a decrease at older ages. U‐shape relationship was also revealed to compensate for the cognition deficit in attention and subcortical related connectivity at late years. (c) The link between middle occipital gyrus and insula, as well as precuneus and cerebellum, exhibited similar changing trends between SNC and FNC across the adult lifespan. Collectively, these results highlight the benefit of lifespan study and provide a precise description of age‐related regional variation and SNC/FNC changes based on a large dataset.

## 1 INTRODUCTION

Human lifespan development is a major topic of interest in neuroscience. The brain maturation process is likely to experience a predominantly genetically determined growth first, followed by a more plastic gene–environment interaction period (Cao, Huang, & He, 2017; Collin & van den Heuvel, 2013; van den Heuvel et al., 2013). Neurodevelopmental trajectories in gray matter (GM) volumetric variations have been extensively studied (Fjell, McEvoy, Holland, Dale, & Walhovd, 2014; Kennedy et al., 2009; Narvacan, Treit, Camicioli, Martin, & Beaulieu, 2017; Terribilli et al., 2011). Consistent GM reduction was primarily located in frontal, temporal, parietal, and insular area (Abe et al., 2008; Farokhian, Yang, Beheshti, Matsuda, & Wu, 2017; Raz & Rodrigue, 2006). However, conflicting results were also observed when using different sample sizes and age ranges. For example, preservation of hippocampus was found when the samples covered a large lifespan (Bagarinao et al., 2018; Grieve, Clark, Williams, Peduto, & Gordon, 2005), whereas atrophy was observed when age effects were evaluated only in older samples (Lemaitre et al., 2005).

Apart from the cerebral alterations, structural or functional connectivity was also revealed to undergo characteristic variations across the lifespan (Cao et al., 2014; Wang, Su, Shen, & Hu, 2012; Yang et al., 2014; Zuo et al., 2010). For example, a number of studies focused on changes in functional network connectivity (FNC) have stated that FNC tended to decrease within resting‐state networks (RSNs) with aging, including visual network and default mode network (DMN; E. A. Allen et al., 2011), and increase between RSNs, especially between components of somatomotor network, ventral attention network and dorsal attention network, which were best fit by convex quadratic models (Betzel et al., 2014). FNC can be extracted from different methodologies, including independent component analysis (ICA) and ROI‐based network construction. In this study, the FNC matrix was estimated by computing the correlation among pairs of time courses identified from ICA as (Calhoun, Adali, Pearlson, & Pekar, 2001; Jafri, Pearlson, Stevens, & Calhoun, 2008; Segall et al., 2012; Xu, Groth, Pearlson, Schretlen, & Calhoun, 2009), which captures networks that co‐vary across time courses. In parallel, we followed our previous paper (Erhardt, Allen, Damaraju, & Calhoun, 2011; Segall et al., 2012) to define the relationships between different structural ICA derived components as structural network correlations (SNC).

Here, leveraging a large dataset (5,967 scans) covering subjects at every age from 13 to 72 years old, we aimed to provide a more robust and precise elaboration of age‐related variations of GM volume (GMV), SNC and FNC. Moreover, based on a well‐matched structure–function template, we compared how age‐related FNC changes were similar to age‐related SNC variations. We performed three analyses in response to the following hypotheses regarding the age‐varying imaging discoveries: (a) As for the GMV, we expected hippocampus or para‐hippocampus would show an inverted U‐shape relationship with age, since this region has been widely reported to be sensitive to aging (Bartsch & Wulff, 2015; Burke et al., 2018). (b) For structural or functional network investigation, we hypothesized that inverted U‐shape relationships would be revealed between FNC or SNC of brain regions in charge of higher‐order cognitive processing, since the late‐maturing brain regions are revealed to be more sensitive to the deleterious effects of aging (Kalpouzos et al., 2009; Toga, Thompson, Mori, Amunts, & Zilles, 2006; Zuo et al., 2017). (c) An exploratory analysis: after examining the age‐varying FNC and SNC patterns, we compared between each other and expected to find certain similarity between functional and structural links.

## 2 MATERIALS AND METHODS

### 2.1 Data acquisition and preprocessing

The data used in this study consisted of 6,101 structural magnetic resonance imaging (MRI) scans and 7,500 resting‐state functional MRI (fMRI) scans, which were collected at the University of New Mexico (UNM) and the University of Colorado Boulder (UC, Boulder). Data in the UC, Boulder site were collected using a 3T Siemens TIM Trio MRI scanner with 12 channel radiofrequency coils, while data in UNM site were acquired using the same type of 3T Siemens TIM Trio MRI scanner, and a 1.5T Avanto MRI scanner. For the scans, MRI protocols were harmonized for all subjects. Site effects were controlled for in the subsequent analysis. All the data were previously collected, anonymized, and had informed consent received from subjects. As the data is a de‐identified convenience dataset, we do not have access to the health and identifier information. Some individuals with brain disorders were likely included, however, we have confirmed that the brain images do not have any obvious pathology or atrophy.

T1‐weighted structural images were acquired with a five‐echo MPRAGE sequence with TE = 1.64, 3.5, 5.36, 7.22, 9.08 ms, TI = 1.2 s, TR = 2.53 s, flip angle = 7°, number of excitations = 1, field of view = 256 mm, slice thickness = 1 mm, and resolution = 256 × 256. The structural data were preprocessed based on voxel‐based morphometry (VBM) in SPM12 (Ashburner & Friston, 2005). The preprocessing pipeline included: (a) spatial registration to a reference brain; (b) joint bias correction and tissue classification into GM, white matter and cerebrospinal fluid using SPM12 old segmentation; (c) spatial normalization to the standard Montreal Neurological Institute (MNI) space using nonlinear transformation; (d) modulation by scaling with the amount of volume changes, and (e) smoothing to 10 × 10 × 10 mm FWHM (Silver et al., 2011; Sui et al., 2013). The smoothed GMV images from each dataset were spatially correlated to the mean image to assess outliers. Scans with a correlation <0.7 were removed.

The fMRI images were used in a previous study that evaluated replicability in time‐varying functional connectivity patterns (Abrol et al., 2017), which has clearly reported the acquisition parameters and preprocessing pipelines. T2*‐weighted functional images were acquired using a gradient‐echo EPI sequence with TE = 29 ms, TR = 2 s, slice thickness = 3.5 mm, flip angle = 75°, slice gap = 1.05 mm, matrix size = 64 × 64, field of view = 240 mm, voxel size = 3.75 mm × 3.75 mm × 4.55 mm. The data preprocessing pipeline included discard of the first three images for the magnetization equilibrium, realignment using INRIalign (Freire & Mangin, 2001), timing correction with the middle slice as reference, spatial normalization into the MNI space. Images collected at 3.75 mm × 3.75 mm × 4.55 mm were then slightly upsampled to 3 mm × 3 mm × 3 mm, resulting in a data cube of 53 × 63 × 46 voxels. The upsampled images were further smoothed with a 10 mm Gaussian model (Silver et al., 2011). The fMRI data covered the entire cerebellum. Anomaly detection in the form of correlation analysis on the five upper and lower slices of the functional images was performed on all 7,500 scans in order to detect scans that failed the reorientation process or had any missing slices. This outlier detection removed 396 subjects, thus leaving behind a total number of 7,104 subjects corresponding to approximately 95% of the available data. The time courses for all subjects were postprocessed in the FNC construction step to remove any residual noise sources.

After preprocessing, 5,967 scans were retained with both structural and functional MRI images. The complete demographic information was shown in Table 1.

**Table 1:** Demographic information

	Numbers of subjects
Total	5,967
Gender	
Male	3,757
Female	2,210
Site	
Site 1 (UC Boulder 3T)	490
Site 2 (UNM 3T)	3,914
Site 3 (UNM 1.5T)	1,563
Age (y)	
13–22	2,080
23–32	1,690
33–42	934
43–52	710
53–62	364
63–72	189

### 2.2 Independent components derived from ICA for functional and structural data

ICA analysis on the functional data was conducted in our previous study (Abrol et al., 2017) using group ICA (GICA) implemented in the GIFT toolbox (http://mialab.mrn.org/software/gift/; Calhoun, Adali, Pearlson, & Pekar, 2002). The number of components was set to be 100. After visual inspection of all the 100 components, 61 components were selected with peak activations in GM, time courses dominated by low‐frequency fluctuations, and high spatial overlap with resting networks. We then grouped the 61 components into a nine‐network template: visual network (VIS), somatomotor network (SM), dorsal attention network (DA), ventral attention network (VA), limbic network (LIMBIC), frontoparietal network (FP), DMN, subcortical network (SUB), and cerebellar network (CB). The first seven networks were identified through quantitative comparisons with Yeo et al.'s seven‐network template (Yeo et al., 2011). We extended to include subcortical and cerebellar regions as two additional networks, which were identified using the anatomical automatic labeling (AAL) template. The criteria for sorting the components was based on the peak location.

**Figure 1:**
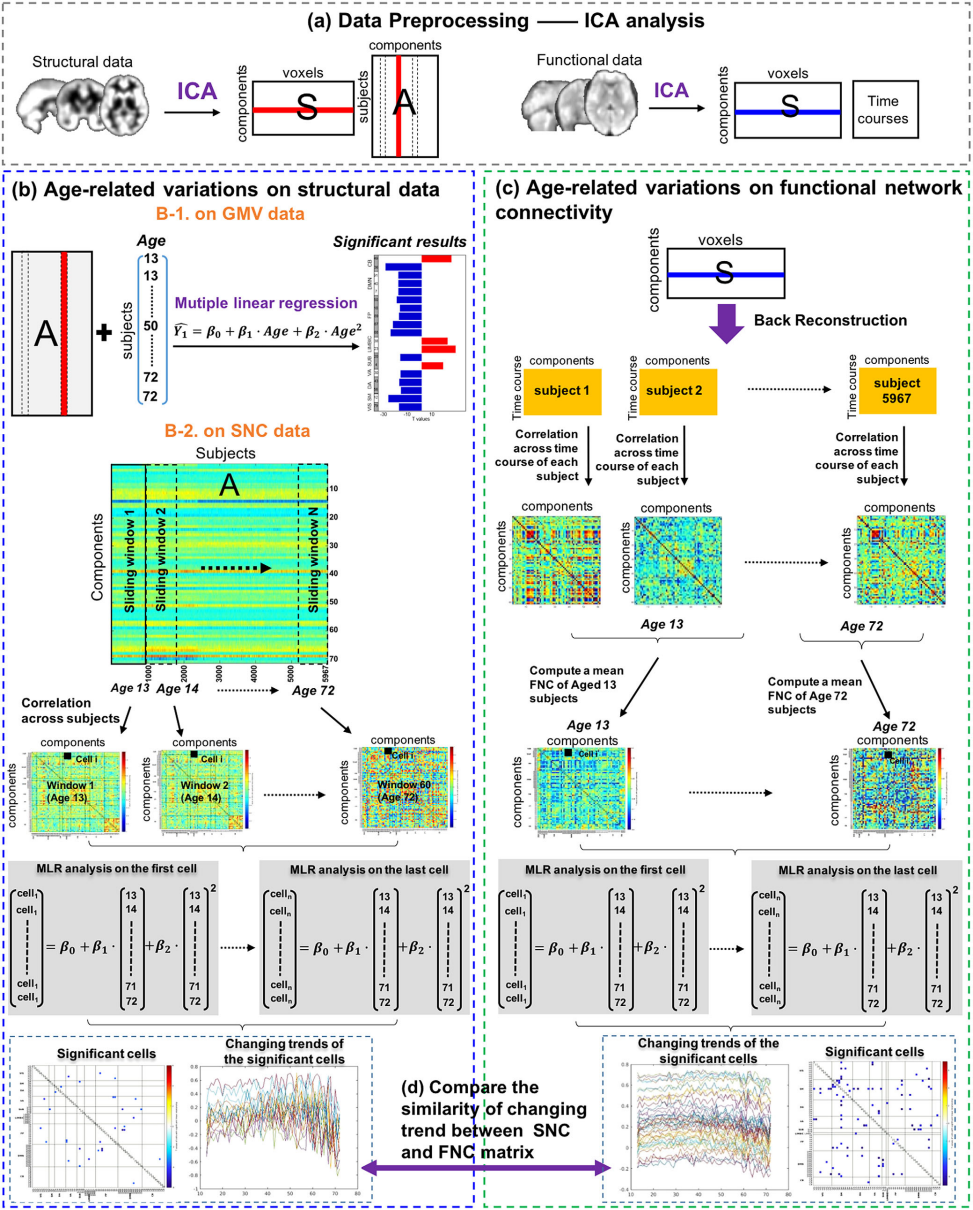
Illustration of the analysis pipeline. (a) Structural and functional data were first decomposed by ICA. (b) The analysis pipeline of computing the relationship between age and structural data. (b‐1) We first computed the relationship between each component in the A matrix and age using a multiple linear regression (MLR) model, which measures how GMV changes across the adult lifespan. (b‐2) We then applied a sliding‐age window to the structural loading parameters [A matrix] to construct structural network correlation (SNC) matrix for each age stage and further examined the relationship between age and SNC using MLR. (c) For the functional data, group‐level spatial maps [S] were used to back reconstruct the matrix [time courses by components] for each subject and then functional network connectivity (FNC) was constructed crosstime courses for each subject. We further computed the mean FNC matrix for each age stage and investigated the significantly age‐related cells across all mean FNC matrices using the same MLR model. (d) Based on the significantly age‐related SNC and FNC cells, we finally measured the similarity of the changing trends for all the paired SNC cells and FNC cells identified from a well‐matched structure–function template

ICA decomposition on the structural data was investigated with source‐based morphometry (SBM), which decomposed the GMV images into a loading parameter matrix (the A matrix in Figure 1a) and a source matrix (the S matrix in Figure 1a; Calhoun et al., 2001; Xu et al., 2009). The loading parameter matrix represented the weight of components for each subject and the source matrix indicated the corresponding spatial maps. For the purpose of comparing the similarities between age‐related structural and functional changes, we used the same number of components (100) as functional data for ICA analysis. Components with significant spatial overlap with ventricles, large vasculature, white matter and the brainstem, or located at the boundaries between these regions and GM were excluded as (Du et al., 2015). Of all the 100 structural components identified from ICA, 71 GM components were retained for analysis after removal of artifact components via visual inspections and further divided into the nine domains defined above (Figure S2).

### 2.3 Construction of age‐resolved SNC and FNC matrix

#### 2.3.1 Network construction from structural data

**Figure 2:**
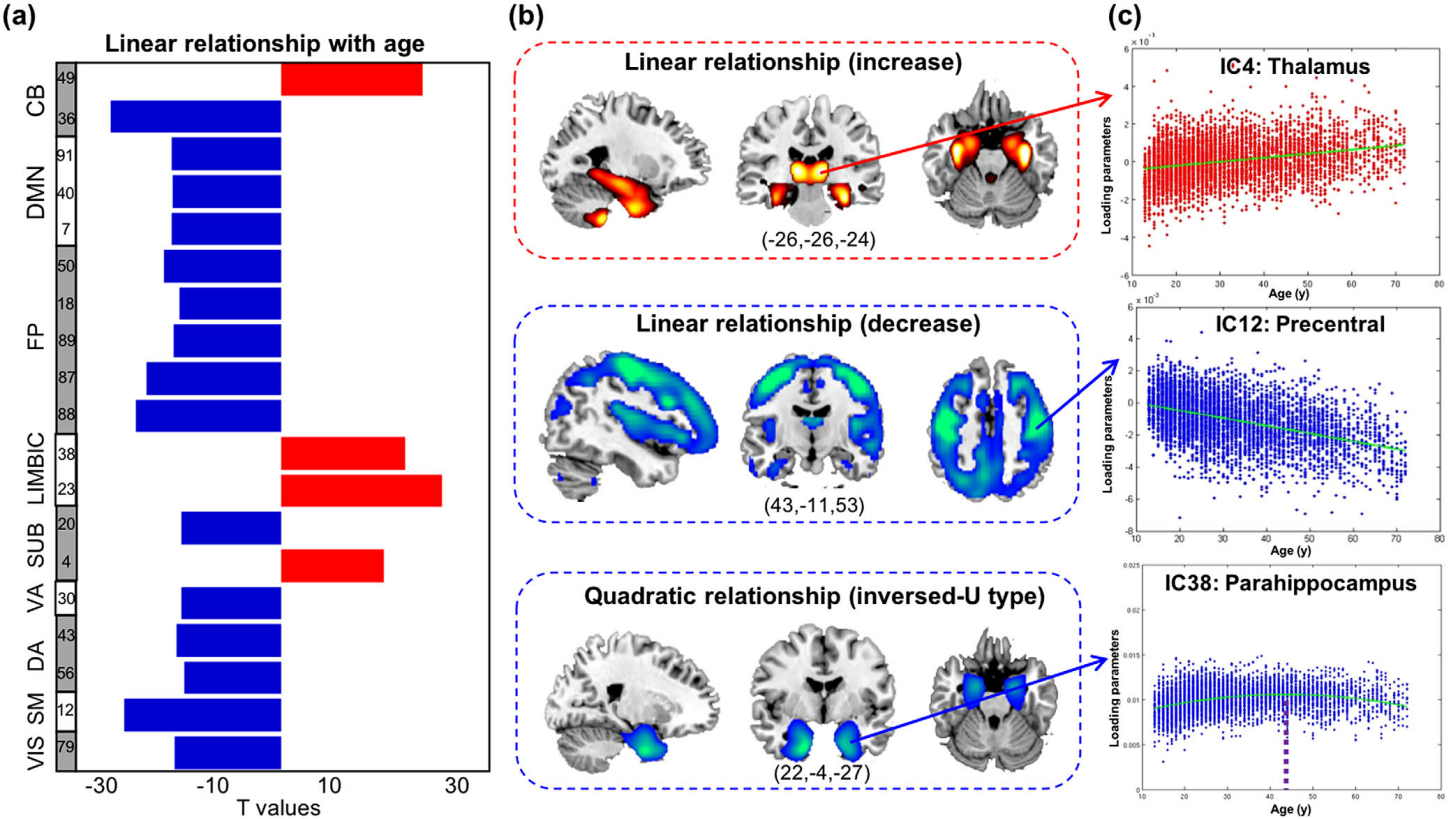
The relationships between age and gray matter volume. (a) T values of the significant components. (b) Spatial maps of the significant components; three types of relationship were revealed. (c) Scatter plots of the representative components in each type. The blue color indicated negative T values for all relationships

SNC matrices were constructed from 13 to 72 years old using a sliding‐age window method **(**Figure 1b‐2**)**. Loading parameters were cross‐correlated within windows that contained participants of the same age and incrementally moved across the age‐range in regular increments (Vasa et al., 2018). The step size was set by 1‐year‐old. The window width depended on the number of subjects in each age stage. A partial correlation, using gender, site, and age × gender as covariates, was used to compute the SNC, then 60 SNC matrices would be constructed corresponding to the 60 age stages.

#### 2.3.2 Network construction from functional data

Back reconstruction using group information guided ICA (GIG‐ICA) was performed after GICA (Du & Fan, 2013), with the selected 61 components as a reference, to estimate subject‐level time courses and maps for each subject. The back‐reconstructed time‐courses went through additional processing steps to remove any residual noise sources mostly including low‐frequency trends originating from the scanner drift, motion‐related variance emerging from spatial nonstationarity caused by movement, and other nonspecific noise artifacts unsatisfactorily decomposed by the implemented linear mixed model. More specifically, the postprocessing steps featured de‐trending existing linear, quadratic, and cubic trends, multiple linear regression of all realignment parameters together with their temporal derivatives, outlier detection using 3D spike removal, and low pass filtering with high‐frequency cut‐off being set to 0.15 Hz. Finally, the time courses were variance normalized. An FNC matrix was finally constructed across time courses for each subject with gender, site and age × gender as covariates (Jafri et al., 2008). After that, we sorted all FNC matrices into an age‐increasing order and computed a mean FNC matrix for each age stage (from 13 to 72 years old; Figure 1d). Accordingly, 60 FNC matrices were constructed corresponding to 60 age stages.

### 2.4 Correlations between age and GMV, SNC, and FNC

We applied a following multivariate linear regression (MLR) model to compute the relationship between age and GMV (case 1: Figure 1b‐1), SNC (case 2: Figure 1b‐2) and FNC (case 3: Figure 1c).

(1) $$\hat{Y}=\beta_{0}+\beta_{1}∙\mathrm{Age}+\beta_{2}∙{\mathrm{Age}}^{2}+\beta_{3}∙\text{covariates}+e$$

Here, $\hat{Y}$ is a [5,967 × 1] vector, representing the structural loading parameters for case 1. $\hat{Y}$ is a [60 × 1] vector, representing the values of the same cell in all 60 SNC/FNC matrix for case 2 and case 3. Age term includes ages with associated parameters *β*_1_. Age^2^ term is a matrix consisting of squared ages with the associated parameter *β*_2_, and *e* is an error term. In case 1, covariates term indicated gender, site and age × gender. As these covariates were already regressed when constructing the SNC and FNC matrices, the covariates term was crossed out for case 2 and case 3. Cells with significant *p* values (FDR < 0.05) of parameter *β*_1_ indicated an age‐related linear relationship, and cells with significant *p* values (FDR < 0.05) of parameter *β*_2_ indicated an age‐related quadratic relationship. In case 1, as 71 structural components were used to measure the relationship between structural loading parameters and age using the MLR model, FDR correction for the significance of *β*_1_ and *β*_2_ was based on the 71 *p* values. In case 2, as the MLR model was used to select the significant cells in the SNC matrices across 60 age stages, FDR correction for the significance of *β*_1_ and *β*_2_ was based on 2,485 cells ([71×70]/2). In case 3, FDR correction for the significance of *β*_1_ and *β*_2_ was based on 1830 cells ([61×60]/2). At last, T maps corresponding to these significant cells were further plotted.

### 2.5 Comparisons between age‐related SNC and FNC variations

To further compare similarities between age‐related SNC changes and FNC variations, we adopted a well‐matched structure–function template revealed in a recent study (Luo et al., 2019, Figure S3 **)**. Based on the template, we could find the matching age‐related cells between SNC and FNC matrix. Then we plotted curves with age of these matched cells and further computed the correlation between curves of each matched pair **(**Figure 1e**)**.

## 3 RESULTS

### 3.1 GMV changes across the adult lifespan

Nineteen GM components were revealed to show a linear relationship with age (significance was measured using effect magnitude [partial *R* ^2^ > .05] and FDR‐corrected *p* values [*p* < 0.05]). Among these, 15 components exhibited a linear declining relationship **(**Figure 2a**)**. Components from cerebellar and somatomotor domains, especially the vermis and precentral area, showed the highest declining correlations with age **(**Figure 2b**)**. Four other components (s‐IC4, s‐IC23, s‐IC38, and s‐IC49) exhibited a linear positive relationship with age, which were composed of the thalamus, parahippocampal, hippocampus, and parts of the cerebellum, respectively. The component that primarily consisted of the parahippocampus and temporal pole (s‐IC38) further exhibited an inverted U‐shape relationship with age. By fitting the component into a quadratic plot, we estimated the turning point to be 43.68 years old as shown in Figure 2c.

### 3.2 SNC changes across the adult lifespan

**Figure 3:**
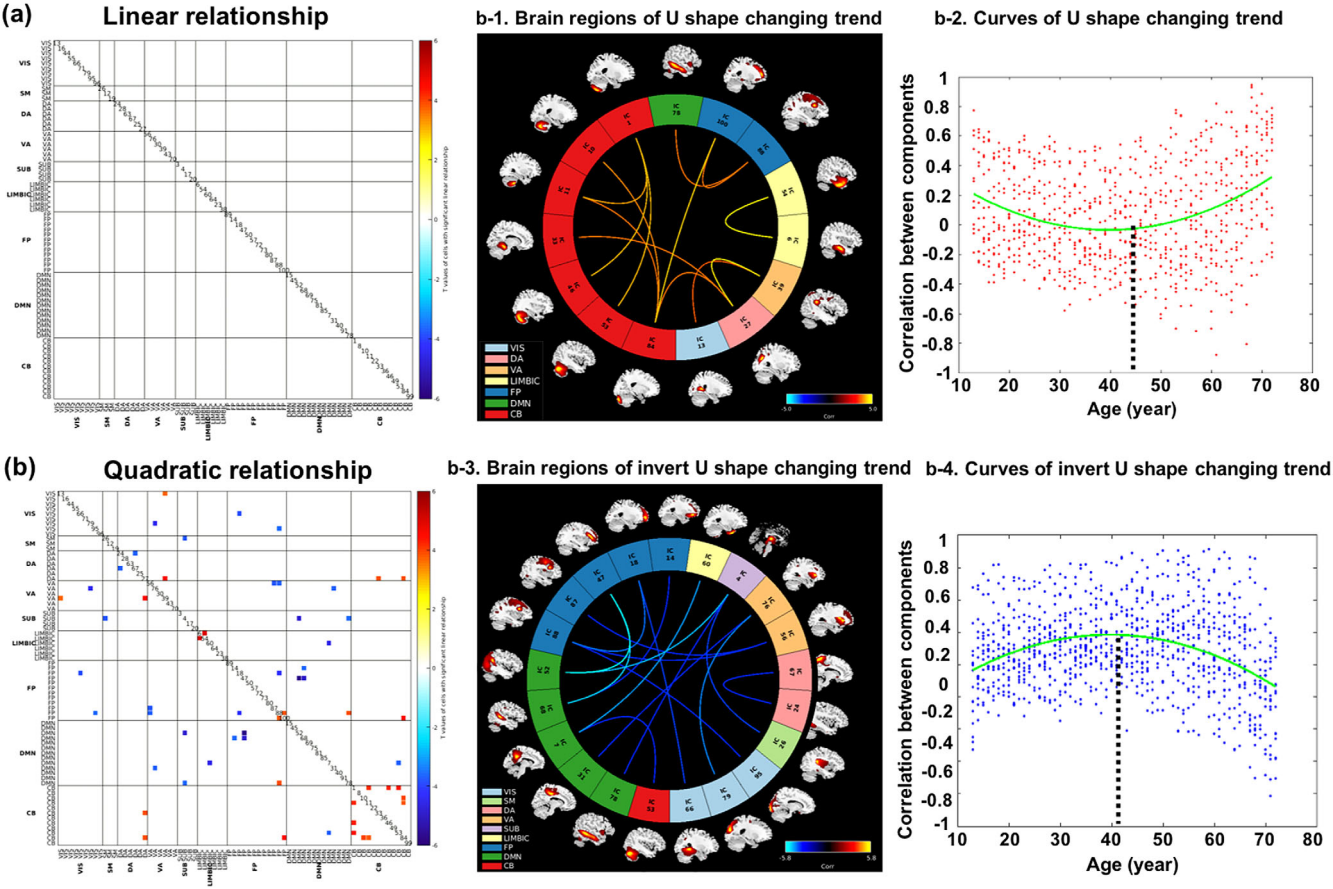
Correlations between age and structural network correlation. (a) No significant linear relationship was revealed. (b) Both U‐shape and inverted U‐shape quadratic relationship were revealed. Figure 3b‐1,b‐2 separately depict the connectome of the significant U‐shape cells and the changing trends across the adult lifespan. Figure 3b‐3,b‐4 separately depict the connectome of the significant inverted U‐shape cells and the changing trends across the adult lifespan

**Figure 4:**
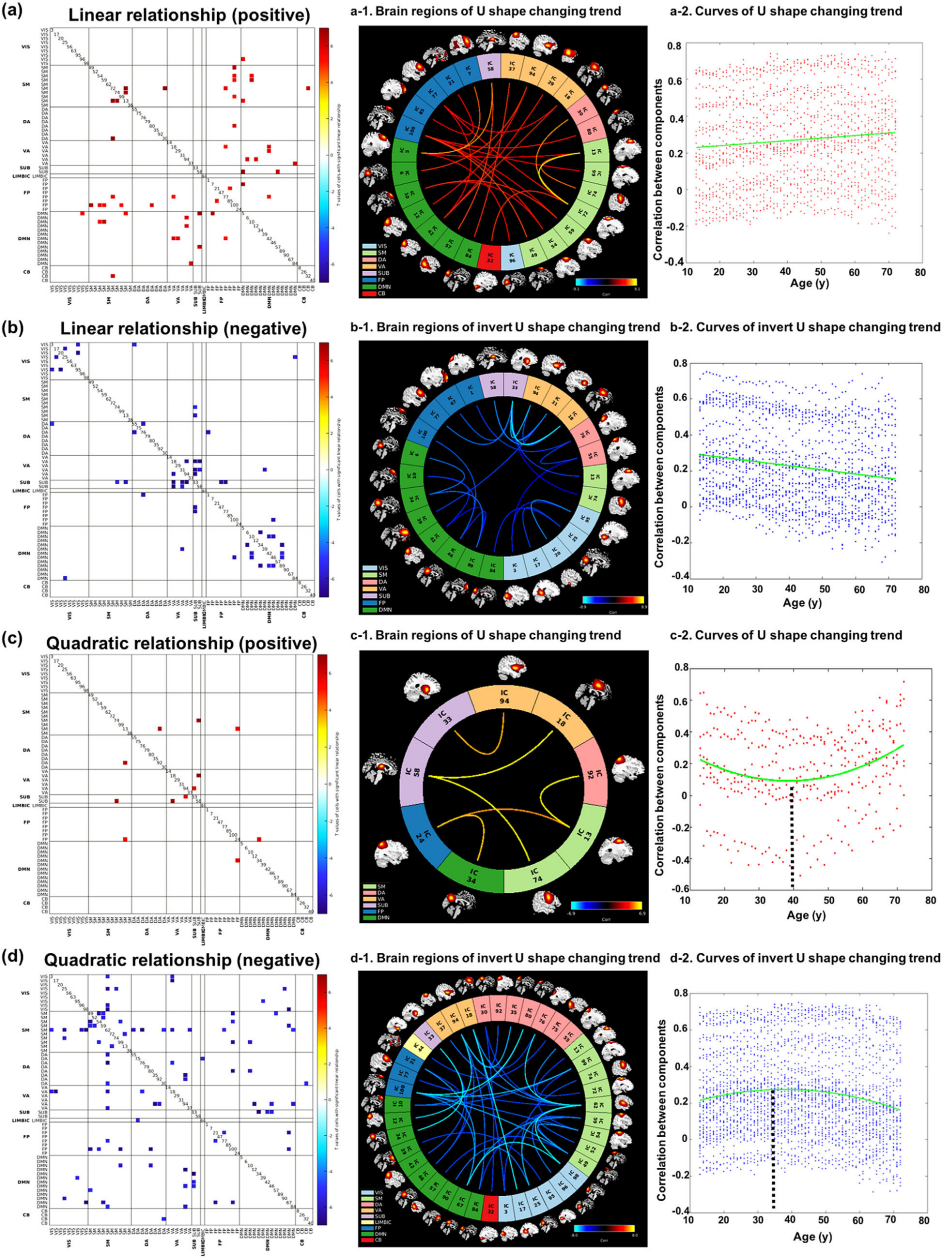
The relationship between age and functional network connectivity. (a) Cells exhibiting a linear positive relationship with age; (a‐1): the connectome of the significant cells; (a‐2): the changing trends of the significant cells. (b) Cells exhibiting a linear negative relationship with age; (b‐1): the connectome of the significant cells; (b‐2): the changing trends of the significant cells. (c) Cells indicating a U‐shape relationship across the adult lifespan; (c‐1): the connectome of the significant cells; (c‐2): the changing trends of the significant cells. (d) Cells showing an inverted U‐shape relationship across the adult lifespan; (d‐1): the connectome of the significant cells; (d‐2): the changing trends of the significant cells

Figure 3a,b indicate the significant cells showing linear and quadratic SNC changes with age. No cells presented significant linear relationship with age, while quadratic relationship (FDR < 0.05) were revealed, including both U‐shape (13 cells) and inverted U‐shape types (16 cells). To rule out randomness, we further computed the pairwise correlation between the 13 U‐shape cells and 16 inverted U‐shape cells. As shown in Figure S4, among all 208 pairs of cells, 96 pairs presented significant correlation after FDR correction (FDR < 0.05). For each paired cell, we examined the relationship between age and one cell (U or inverted U‐shape) while controlling for the other cell. As shown in Table S1, the majority of the significant cells showing quadratic relationships are retained (light blue background), suggesting that it is unlikely to identify both U and inverted U‐shape relationship by randomness. Most significant cells with a U‐shape relationship were observed within the cerebellar network. The connectomes of the significant cells are plotted in Figure 3b‐1. Figure 3b‐2 describes the changing trends across the adult lifespan for all significant cells. By fitting these scatters into a quadratic plot, the turning point was estimated to be 45.17 years old. Meanwhile, inverted U‐shape relationships were primarily revealed in DMN and FP related correlations. Figure 3b‐3,b‐4 separately depict the connectome of the significant cells and the changing trends across the adult lifespan. By fitting these scatters into a quadratic plot, the turning point was estimated to be 40.83 years old.

### 3.3 FNC variations across the adult lifespan

Figure 4a,b presents the significant FNC cells showing positive and negative linear relationship with age separately (FDR < 0.0001). The FNC cells within RSNs, especially the VIS and DMN domains, linearly decreased with age increase (Figure 4a). Linear increase was revealed between RSNs, primarily in FP and DMN (Figure 4b). The connectomes of the significant cells are plotted in Figure 4a‐1 and Figure 4b‐1. Figure 4a‐2 and Figure 4b‐2 plot the changing trends across the adult lifespan for the significant cells. U‐shape relationships are primarily revealed in some VA and SUB related connectivity as shown in Figure 4c. Inverted U‐shape relationships with age are revealed primarily between RSN, centered on the SM, FP, and DMN networks (Figure 4d, FDR < 0.0001). The connectomes of the significant cells are plotted in Figure 4c‐1,d‐1. Figure 4c‐2,d‐2 separately plot the changing trends of the significant cells. By fitting these scatters into a quadratic plot, the turning points were estimated to be 40 years old for U‐shape and 36.5 years old for inverted U‐shape relationships. To further examine the effect of head motion on our results, we first computed the mean framewise displacements (FD) for each subject (Power, Barnes, Snyder, Schlaggar, & Petersen, 2012; C. G. Yan et al., 2013). The relationship between mean FD and age was not significant as shown in Figure S5 (*r* = 0.0078, *p* = .5485). We then added the mean FD as a covariate to compute the age‐related significant FNC cells again. The results are similar to the raw results (Figure S6). Moreover, as 720 scans were identified with mean FD larger than 0.5 among all 5,967 subjects, we measured the age‐related FNC variations again based on the remaining 5,247 subjects. As shown in Figure S7, the results are highly consistent with the original results, suggesting that head motion does not have much impact on our results in this study.

### 3.4 Comparisons between age‐related SNC and FNC variations

**Figure 5:**
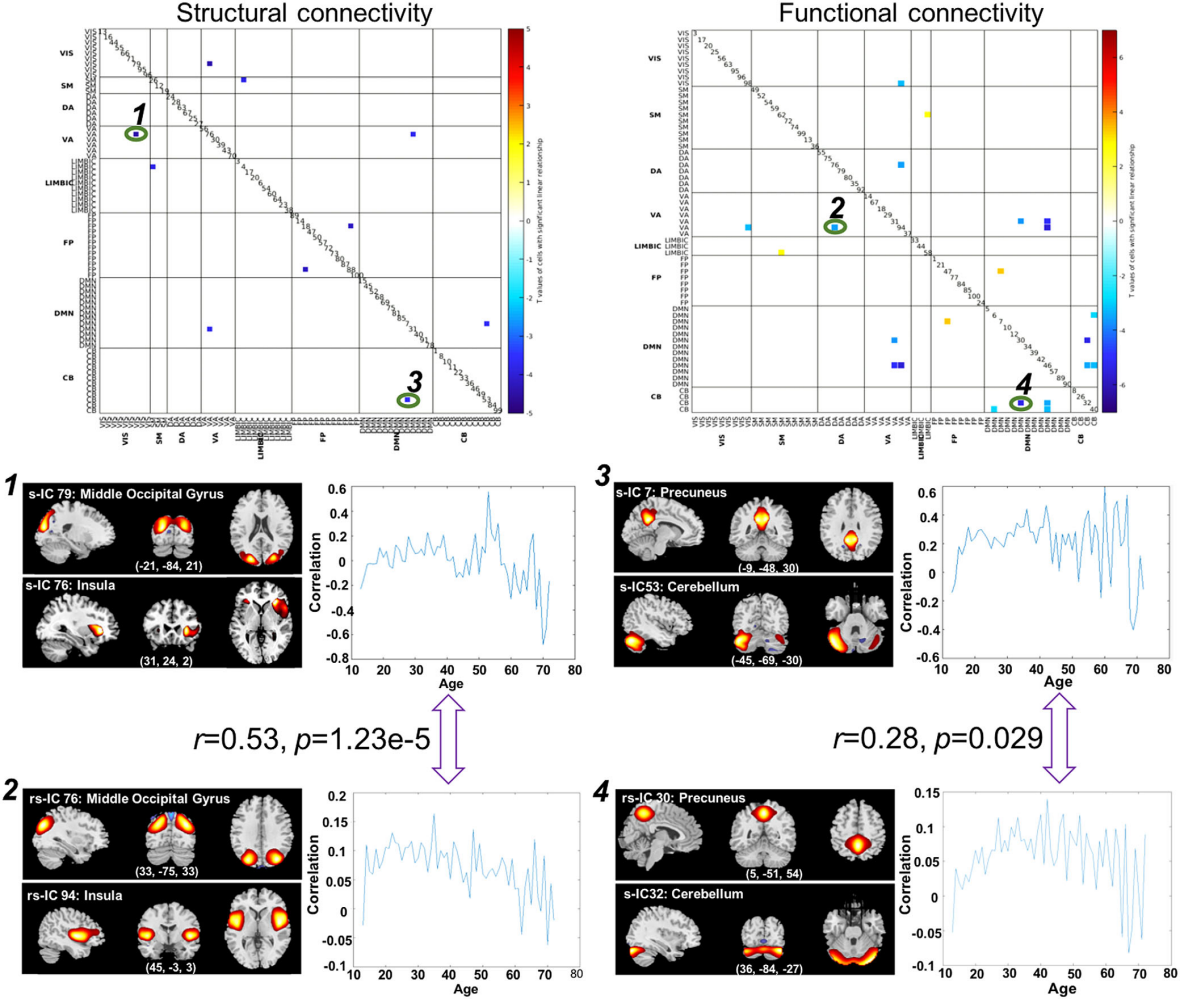
The cells presenting similar inverted U‐shape relationship with age between SNC and FNC. Two cells (green circle) were revealed with the significant similar changing trend: middle occipital gyrus‐insula link (cell 1 and cell 2, *p* = 1.23 × 10^−5^) and precuneus‐cerebellum link (cell 3 and cell 4, *p* = .029)

After applied the well‐matched structure–function template, 5 SNC cells are identified to be matched with 11 FNC cells as shown in Figure 5. Only two overlapping cells exhibited similar inverted U‐shape between SNC and FNC across the adult lifespan. One is the link between the middle occipital gyrus and insula (cell1‐cell2: *r* = 0.53, *p* = 1.23 × 10^−5^), the other one is the link between the precuneus and cerebellum (cell3‐cell4: *r* = 0.28, *p* = .029).

## 4 DISCUSSION

To the best of our knowledge, this is the first study to measure the adult lifespan variation trajectories of GMV, SNC, and FNC based on the year‐wise estimation of a very large dataset (5,967 subjects) from 13 to 72 years old. By applying a well‐matched structure–function template, we further compared how age‐related FNC changes were similar to age‐related SNC variations.

### 4.1 Lifespan changes in GMV

Most trajectories of the structural ICA‐decomposed components showed a linearly declining relationship with age, primarily existed in the SM, DMN and FP network, consistent with previous studies (J. S. Allen, Bruss, Brown, & Damasio, 2005; Liu et al., 2017; Spreng & Turner, 2013). For example, Spreng et al. observed that GMV declined linearly with age in DMN across the adult lifespan of 18–96 years (Spreng & Turner, 2013). Allen et al. revealed a negative correlation between GMV and age in the frontal, parietal and visual networks (J. S. Allen et al., 2005). Relative preservation of GMV in the thalamus and parahippocampal gyrus has been similarly reported as (Bagarinao et al., 2018). An inverted U‐shape relationship was further revealed in parahippocampal area, consistent with (J. S. Allen et al., 2005; Kalpouzos et al., 2009). Notably, we also observed a significant inverted U‐shape relationship in s‐IC23, with peaks at hippocampus and fusiform, however, the partial R square for these components was 0.017. In this study, we only reported the components which presented a partial R square value larger than 0.05. Moreover, selection bias in older participants in cross‐sectional data is a potential limitation to the lower age‐related reduction in the hippocampus of elder subjects as highlighted in (Nyberg et al., 2010). In addition, the GMV showed both positive and negative linear correlations with age in the cerebellar and subcortical networks (Figure 2). As shown in Figure S2, even though both s‐IC49 and s‐IC36 belongs to the cerebellar network, the s‐IC36 composed of the vermis and s‐IC49 primarily composed of the posterior cerebellum. Previous studies have reported different volumetric trajectories in the anterior cerebellum/vermis, and posterior cerebellum. The anterior cerebellum and vermis follow a logarithmic pattern such that volumes were largest in adolescents and dropped quickly during young adulthood (Bernard, Leopold, Calhoun, & Mittal, 2015). While the volumetric pattern of the posterior cerebellum seems to follow the protracted developmental pattern of the prefrontal cortex (Bernard et al., 2015). Moreover, it was suggested that this posterior cerebellar motor representation serves different functions than the anterior cerebellar motor representation (Donchin et al., 2012). Therefore, we would observe different relationship with age in the two areas. For the subcortical network, the s‐IC20 presented negative relationship with age, with peaks at the caudate, while a positive relationship was observed in s‐IC4, which composed of thalamus. Previous studies have also reported different age‐related volumetric variations in caudate (Walhovd et al., 2011) and thalamus (Bagarinao et al., 2018; Grieve et al., 2005).

### 4.2 Lifespan changes in structural network correlation

In order to make an extensive examination of age‐related variations in both cortical properties and connectivity, we also studied how structural or functional connectivity changed with age across the adult lifespan. The DMN and FP related connectivity presented an inverted U‐shape relationship, which are consistent with “last‐in‐first‐out” rule: the late‐maturing brain regions are revealed to be more sensitive to the deleterious effects of aging (Kalpouzos et al., 2009; Terribilli et al., 2011). The connectivity of these areas would mature after other brain areas, followed by atrophy, and then present a significant inverted U‐shape tendency with age. Collin et al. also suggested that the dynamic changes in connectome organization throughout the lifespan follows an inverted U‐shaped pattern (Collin & van den Heuvel, 2013). Moreover, since these brain regions are primarily involved in cognitive functions like attention, executive function and cognitive control, older brain will present increased activation in other related connectivity, for example, the dorsal attention and ventral attention network in this study, to reveal neural compensatory mechanisms (Romero‐Garcia, Atienza, & Cantero, 2014), leading to a U‐shape relationship. Structural correlation within cerebellar network also presented a U‐shape relationship with age, which is consistent with previous studies (Brenhouse & Andersen, 2011; Durston et al., 2001). Besides, since the structural data were constructed based on the ICA‐decomposed GM components, the age‐related GMV variations could reflect some SNC changes to a certain degree. For example, as shown in Figure 2a, the GMV linearly increased with age primarily in cerebellum and limbic system, while the other brain regions showed a linearly decreased relationship with age. Consistent with this, we also observed quadratically increased trends within the cerebellar network and quadratically decreased relationship in other brain networks for the SNC cells as shown in Figure 3b.

### 4.3 Lifespan changes in functional network connectivity

Both linear and quadratic relationships with age were revealed in the FNC cells. Early linearly increase in FNC were primarily observed between RSNs, which experienced decrease at older ages, for example, the connectivity in STG, ACG, MCG, and SFG areas, consistent with previously reported results (Betzel et al., 2014; Geerligs, Renken, Saliasi, Maurits, & Lorist, 2015). According to the “last‐in‐first‐out” hypothesis revealed in the frontal and temporal areas as discussed above, the connectivity between frontal and temporal areas matured after other brain areas, followed by atrophy, and then exhibited an inverted U‐shape tendency with age. The FNC within RSNs decreased linearly over the adult lifespan, especially in DMN and VIS, consistent with other studies (Andrews‐Hanna et al., 2007; Geerligs et al., 2015; Tomasi & Volkow, 2012; L. Yan, Zhuo, Wang, & Wang, 2011). Late increase at older ages was observed in attention and SUB related area. The U/inverted‐U shape relationship may implicate the compensation of human brain connectivity among key networks responsible for the cognition deficit in attention and high executive function during aging (Chen et al., 2018).

### 4.4 Comparisons between changes in SNC and FNC

Relationships between age‐related SNC changes and FNC variations are complex. Several previous studies reported that regions with few or no direct structural connections may exhibit high functional connectivity, which indicates the presence of indirect connections between structure and function (Damoiseaux & Greicius, 2009; Honey et al., 2009). Fjell et al. demonstrated that anatomical alignment of SNC and FNC seemed restricted to specific networks, for example, certain regions of the DMN network, and changes in SNC and FNC were not necessarily strongly correlated (A. M. Fjell et al., 2017). In this study, we observed two matched SNC and FNC cells exhibiting a similar inverted U‐shape across the adult lifespan as shown in Figure 5:(a) the link between middle occipital gyrus and insula; (b) the link between precuneus and cerebellum. These have not been shown previously. The link between middle occipital gyrus and insula was reported to be responsible for face emotion processing (Guo et al., 2015). The precuneus has also been identified to react to fearful faces (Zhao, Zhao, Zhang, Cui, & Fu, 2017) and is part of the extended face‐processing network (Fox, Iaria, & Barton, 2009). Cerebellum was revealed to be implicated in rapidly coordinating information processing, aversive conditioning, and learning the precise timing of anticipatory responses (Auday, Taber‐Thomas, & Perez‐Edgar, 2018). Concerning the integration of contextual body signals in facial emotion perception, previous studies have shown an association with precuneus (contextual integration) and cerebellum (motor resonance; Santamaría‐García et al., 2019). Additionally, the turning point of quadratic relationship for age‐related FNC variations was earlier than the turning point of SNC. FNC was revealed to detect brain activity through measuring variations related to blood flow, which are sensitive to environment changes (Deakin et al., 2008; Lahti, Holcomb, Medoff, & Tamminga, 1995). While SNC measures the links between pairs of co‐varying GM patterns, the deficits of which take time to manifest. These may partly help explain why age‐related FNC variations would present an earlier changing point compare to age‐related SNC changes.

### 4.5 Limitations of the current study

There are several limitations to the current study. The first limitation is the lack of assessment of health status for the individuals included, which may leave a potential effect on the results under different psychiatric, neurological, or other neurodegenerative conditions. As the data included a large sample size with a large age range, we believe the results may be more driven by the common characteristics of age‐related changes. The second limitation is the cross‐sectional nature of the data. Studies which used cross‐sectional subjects may suffer from cohort effects and could not investigate changes over time within subjects compared to longitudinal studies. While longitudinal studies cannot totally replace cross‐sectional studies for some limitations, such as the life expectancy of scanners (Salthouse, 2012). Third, although structural covariance of brain region volumes have been proved to be associated with both structural connectivity and transcriptomic similarity (R. Romero‐Garcia et al., 2018; Yee et al., 2018), it is an indirect, group‐wise measurement to scale the structural connectivity compared to tracking individual white matter fiber connectivity using diffusion magnetic resonance imaging (dMRI). Further work evaluating age‐related variations using dMRI‐based white matter connectivity is needed. Fourth, the resolution of the fMRI images is sub‐optimal (3.75 × 3.75 × 4.55 mm) compared to the newer multi‐band sequences (2–3 mm isotropic). In addition, there are some existing large N datasets, for example, ENIGMA (Favre et al., 2019; Hoogman et al., 2019; van Velzen et al., 2019), 10 K in 1 day (van den Heuvel et al., 2019) and morphometric similarity networks (Seidlitz et al., 2018), which introduce several new network features that can be used to investigate age‐related variations in both healthy as well as disease.

## CONFLICT OF INTEREST

The authors report no biomedical financial interests or potential conflicts of interest.

## Supporting information


**Appendix S1**: Supporting Information

## Data Availability

The structural and functional data used in the present study can be accessed upon request to the corresponding authors.
