# Supplementary material for: Age‐related structural and functional variations in 5,967 individuals across the adult lifespan
Source: Hum Brain Mapp. 2019 Dec 26;41(7):1725–37. doi: 10.1002/hbm.24905 (PMC7267948; doi:10.1002/hbm.24905)
Supplement: Supplementary file 1 — Appendix S1: Supporting Information [file HBM-41-1725-s001.docx]

**Supplementary files**

**Table1.** **A:** The p values of relationship between age and one SNC cell with U shape while controlling for the other paired SNC cell with inverted U shape. **B:** The p values of relationship between age and one SNC cell with inverted U shape while controlling for the other paired SNC cell with U shape.

| 1. Correlation between age and one U shape while controlling for the other cell in each pair | | | | | | | | | | | |
| --- | --- | --- | --- | --- | --- | --- | --- | --- | --- | --- | --- |
|  | **P value** |  | **P value** |  | **P value** |  | **P value** |  | **P value** |  | **P value** |
| **pair1** | 3.54E-04 | **pair17** | 3.74E-03 | **pair33** | 5.73E-03 | **pair49** | 3.46E-02 | **pair65** | 1.33E-03 | **pair81** | 1.50E-02 |
| **pair2** | 6.96E-03 | **pair18** | 1.87E-02 | **pair34** | 3.60E-04 | **pair50** | 1.34E-03 | **pair66** | 5.67E-03 | **pair82** | 2.38E-03 |
| **pair3** | 7.00E-04 | **pair19** | 4.15E-04 | **pair35** | 1.79E-02 | **pair51** | 1.02E-02 | **pair67** | 8.23E-03 | **pair83** | 1.46E-02 |
| **pair4** | 9.58E-04 | **pair20** | 2.01E-03 | **pair36** | 7.19E-03 | **pair52** | 3.87E-03 | **pair68** | 1.80E-03 | **pair84** | 1.33E-03 |
| **pair5** | 7.00E-04 | **pair21** | 8.16E-03 | **pair37** | 1.73E-04 | **pair53** | 2.01E-02 | **pair69** | 5.18E-03 | **pair85** | 2.11E-03 |
| **pair6** | 1.56E-02 | **pair22** | 1.89E-03 | **pair38** | 1.07E-02 | **pair54** | 6.49E-03 | **pair70** | 5.25E-02 | **pair86** | 3.57E-02 |
| **pair7** | 6.81E-02 | **pair23** | 2.83E-03 | **pair39** | 4.68E-03 | **pair55** | 9.63E-04 | **pair71** | 1.95E-03 | **pair87** | 5.33E-03 |
| **pair8** | 8.92E-02 | **pair24** | 3.51E-04 | **pair40** | 5.69E-03 | **pair56** | 6.42E-04 | **pair72** | 3.97E-04 | **pair88** | 1.43E-02 |
| **pair9** | 4.25E-04 | **pair25** | 9.46E-03 | **pair41** | 6.35E-03 | **pair57** | 3.82E-02 | **pair73** | 8.87E-02 | **pair89** | 2.75E-03 |
| **pair10** | 5.52E-04 | **pair26** | 2.36E-04 | **pair42** | 3.66E-03 | **pair58** | 2.49E-03 | **pair74** | 7.22E-03 | **pair90** | 7.75E-03 |
| **pair11** | 1.11E-02 | **pair27** | 1.93E-03 | **pair43** | 2.50E-03 | **pair59** | 1.86E-03 | **pair75** | 6.01E-02 | **pair91** | 2.81E-02 |
| **pair12** | 3.59E-02 | **pair28** | 4.26E-03 | **pair44** | 1.42E-04 | **pair60** | 1.15E-03 | **pair76** | 4.19E-02 | **pair92** | 6.65E-03 |
| **pair13** | 6.10E-03 | **pair29** | 4.15E-04 | **pair45** | 1.74E-03 | **pair61** | 5.58E-03 | **pair77** | 1.31E-02 | **pair93** | 1.50E-03 |
| **pair14** | 6.11E-04 | **pair30** | 3.30E-03 | **pair46** | 1.94E-03 | **pair62** | 4.05E-02 | **pair78** | 1.35E-03 | **pair94** | 1.71E-02 |
| **pair15** | 4.09E-03 | **pair31** | 4.16E-03 | **pair47** | 9.38E-03 | **pair63** | 5.44E-03 | **pair79** | 9.17E-03 | **pair95** | 3.44E-03 |
| **pair16** | 5.33E-02 | **pair32** | 9.72E-04 | **pair48** | 1.51E-02 | **pair64** | 8.71E-03 | **pair80** | 9.19E-04 | **pair96** | 1.43E-03 |
| **B.** Correlation between age and one invert U shape while controlling for the other cell in each pair | | | | | | | | | | | |
|  | **P value** |  | **P value** |  | **P value** |  | **P value** |  | **P value** |  | **P value** |
| **pair1** | 3.65E-03 | **pair17** | 5.45E-02 | **pair33** | 7.23E-03 | **pair49** | 4.81E-02 | **pair65** | 8.93E-03 | **pair81** | 3.24E-04 |
| **pair2** | 6.19E-03 | **pair18** | 2.29E-02 | **pair34** | 5.98E-03 | **pair50** | 3.86E-02 | **pair66** | 1.16E-03 | **pair82** | 2.72E-03 |
| **pair3** | 3.51E-02 | **pair19** | 2.51E-02 | **pair35** | 2.87E-02 | **pair51** | 2.63E-02 | **pair67** | 3.03E-03 | **pair83** | 3.61E-04 |
| **pair4** | 2.94E-03 | **pair20** | 6.81E-03 | **pair36** | 1.04E-02 | **pair52** | 8.84E-03 | **pair68** | 6.20E-04 | **pair84** | 2.27E-04 |
| **pair5** | 1.13E-03 | **pair21** | 7.31E-03 | **pair37** | 1.29E-02 | **pair53** | 4.30E-02 | **pair69** | 1.59E-03 | **pair85** | 1.65E-04 |
| **pair6** | 2.09E-03 | **pair22** | 3.59E-02 | **pair38** | 1.39E-02 | **pair54** | 1.73E-02 | **pair70** | 1.92E-02 | **pair86** | 1.10E-03 |
| **pair7** | 7.55E-03 | **pair23** | 4.24E-03 | **pair39** | 7.74E-03 | **pair55** | 1.71E-02 | **pair71** | 2.01E-03 | **pair87** | 1.12E-04 |
| **pair8** | 1.23E-02 | **pair24** | 2.87E-02 | **pair40** | 7.09E-02 | **pair56** | 6.33E-04 | **pair72** | 5.77E-06 | **pair88** | 1.59E-02 |
| **pair9** | 4.48E-04 | **pair25** | 1.65E-02 | **pair41** | 8.46E-03 | **pair57** | 2.94E-03 | **pair73** | 8.54E-05 | **pair89** | 6.83E-02 |
| **pair10** | 1.51E-02 | **pair26** | 2.61E-03 | **pair42** | 5.24E-03 | **pair58** | 1.12E-02 | **pair74** | 4.07E-04 | **pair90** | 1.60E-02 |
| **pair11** | 2.88E-02 | **pair27** | 8.86E-03 | **pair43** | 5.48E-03 | **pair59** | 4.78E-04 | **pair75** | 6.96E-05 | **pair91** | 4.89E-02 |
| **pair12** | 7.93E-02 | **pair28** | 9.89E-03 | **pair44** | 2.91E-04 | **pair60** | 3.37E-04 | **pair76** | 1.48E-04 | **pair92** | 1.44E-02 |
| **pair13** | 1.63E-02 | **pair29** | 6.58E-03 | **pair45** | 2.46E-04 | **pair61** | 7.15E-04 | **pair77** | 8.08E-05 | **pair93** | 2.21E-02 |
| **pair14** | 1.07E-02 | **pair30** | 4.50E-03 | **pair46** | 4.38E-04 | **pair62** | 5.44E-02 | **pair78** | 2.69E-06 | **pair94** | 1.11E-01 |
| **pair15** | 3.01E-02 | **pair31** | 5.13E-03 | **pair47** | 5.69E-04 | **pair63** | 1.82E-01 | **pair79** | 1.24E-04 | **pair95** | 2.31E-02 |
| **pair16** | 3.43E-02 | **pair32** | 1.02E-02 | **pair48** | 5.46E-04 | **pair64** | 1.76E-02 | **pair80** | 2.42E-04 | **pair96** | 1.52E-02 |

**
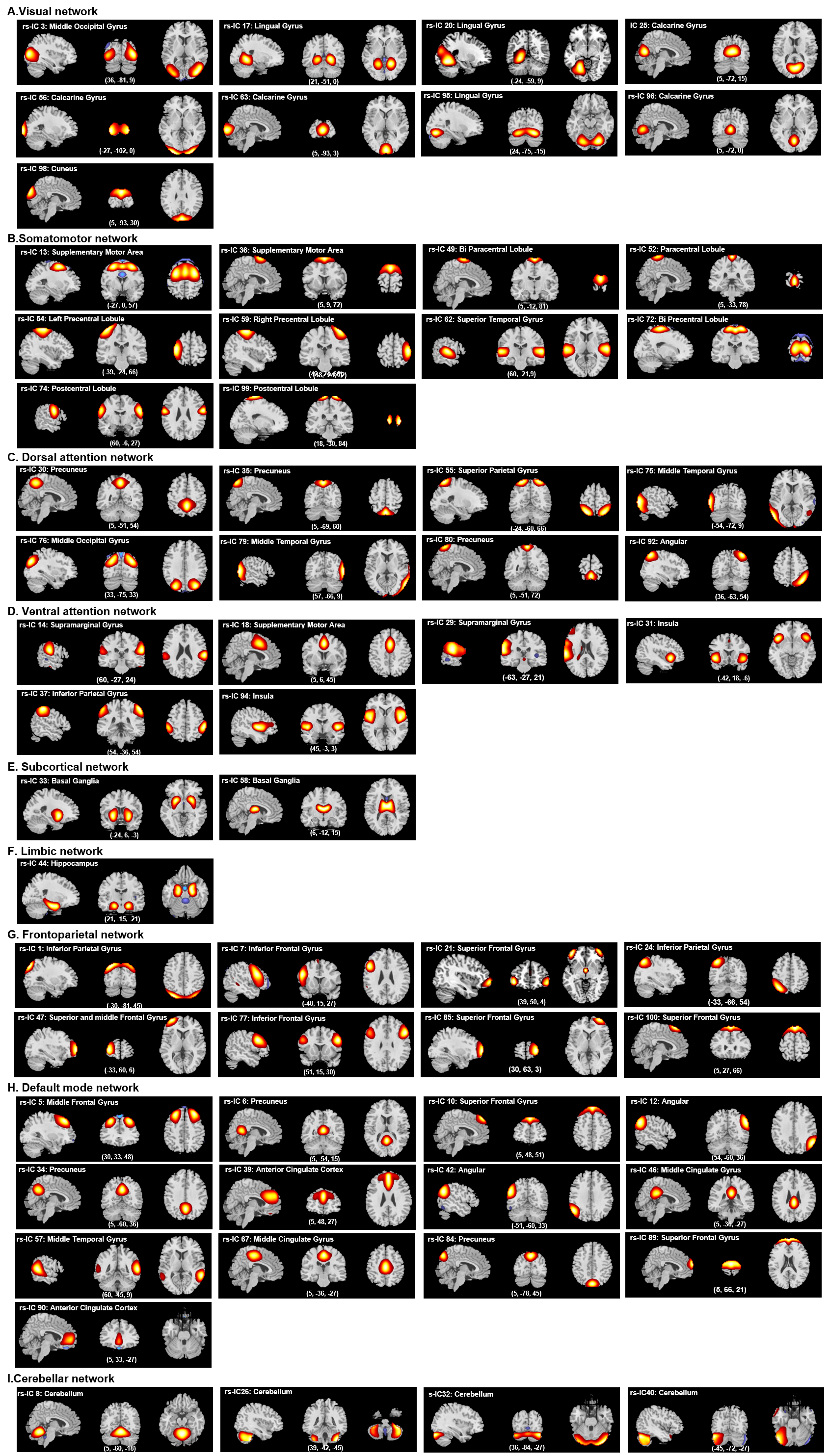
**

**Figure S1.** The selected 61 functional components which were decomposed from Group ICA and sorted into nine domains.

**
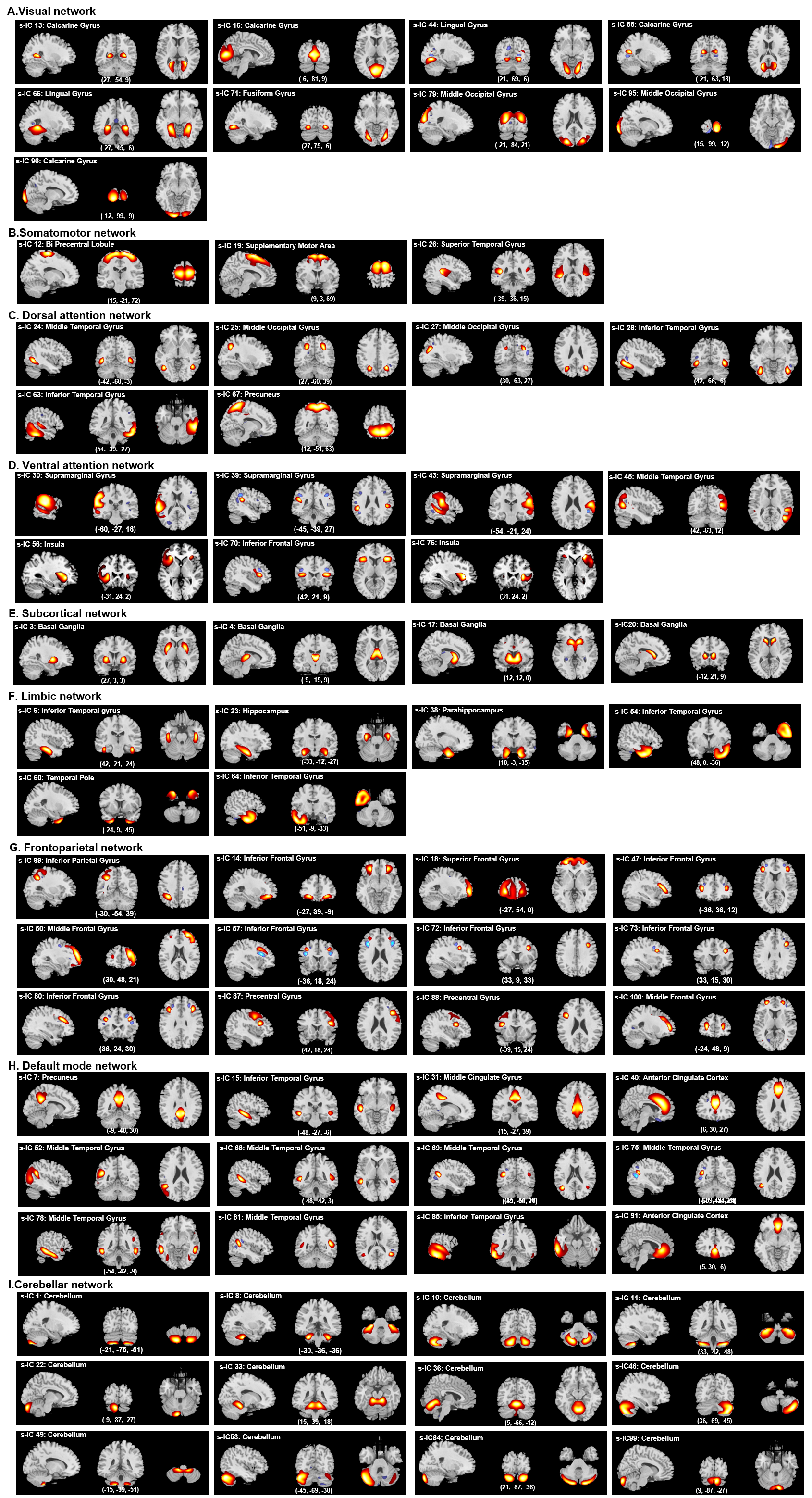
**

**Figure S2.** The selected 71 structural components which were decomposed from ICA and sorted into nine domains.

**
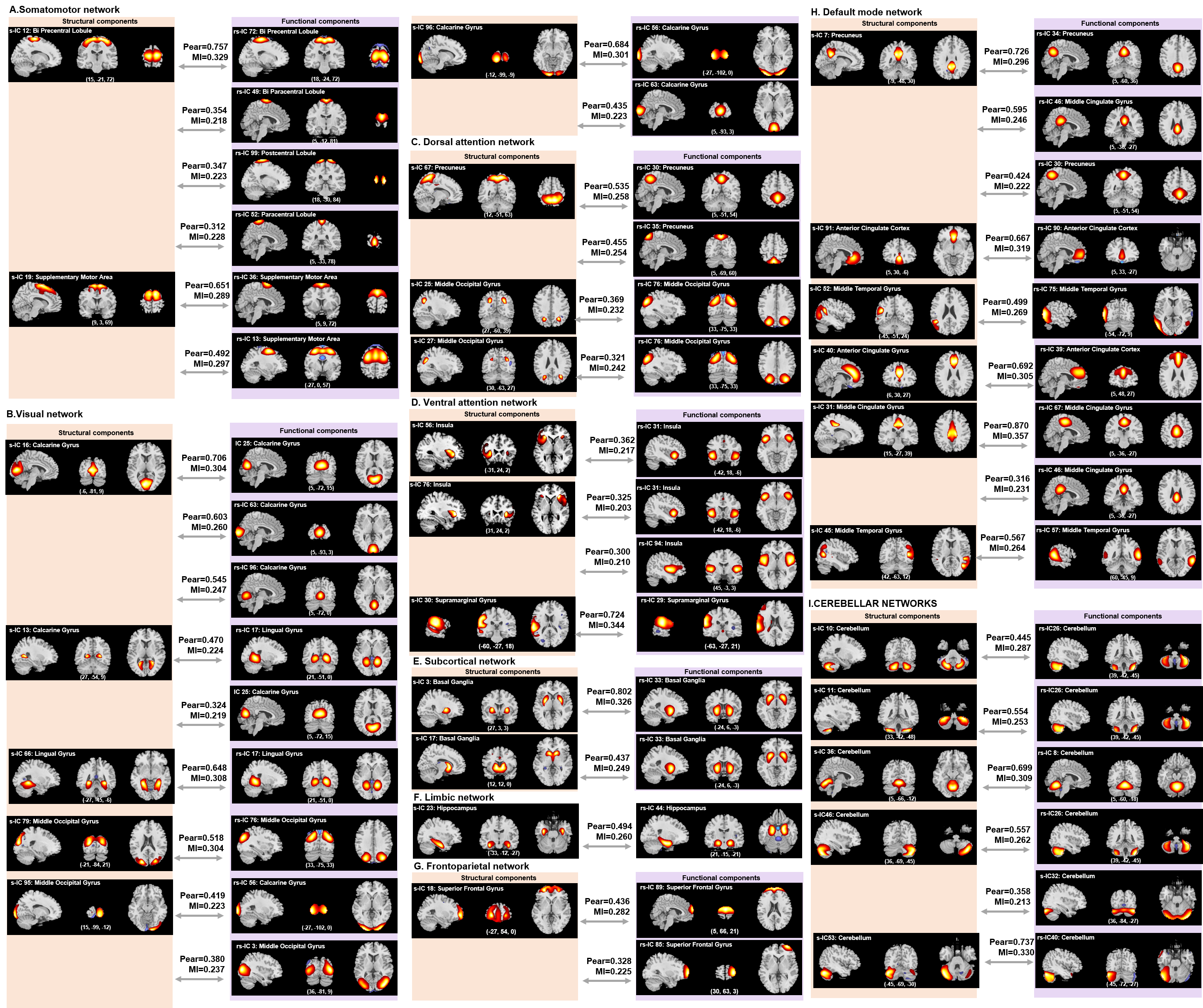
**

**Figure S3.** The well-matched structure-function template.


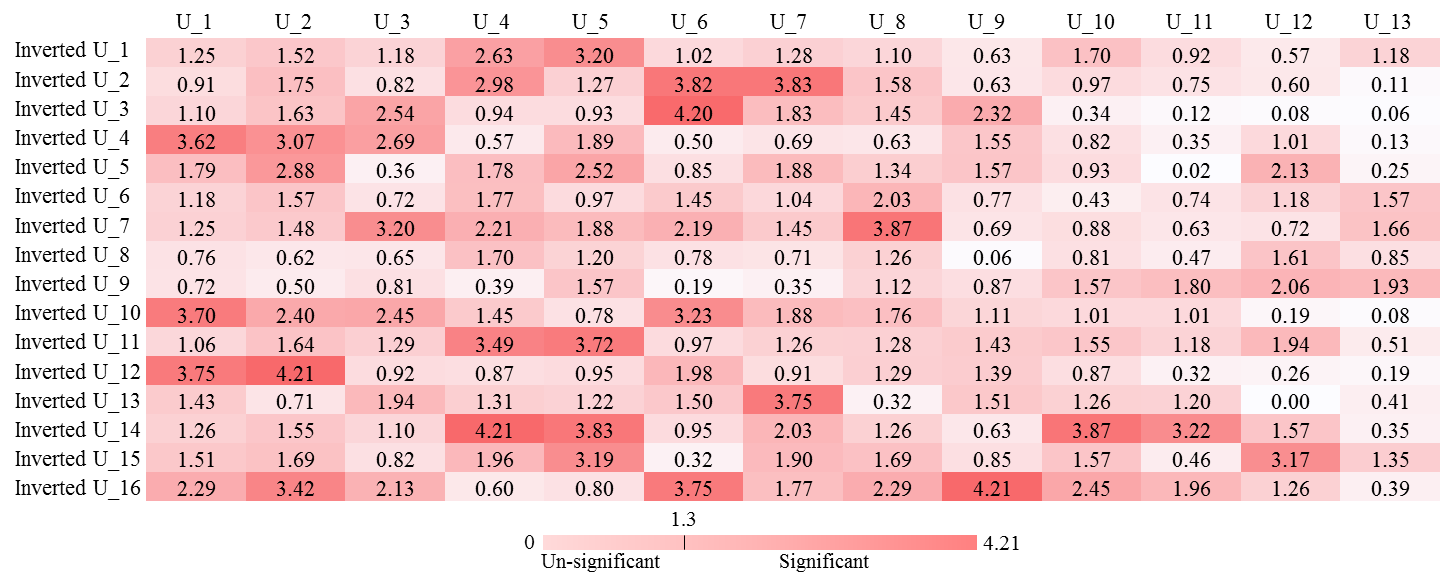


**Figure S4.** Correlation [-log10(fdr_p)] between the 13 U-shape cells and the 16 inverted U-shape cells. Note U_1~13 present the 13 U-shape cells and Inverted U_1~13 present the 16 inverted U-shape cells.


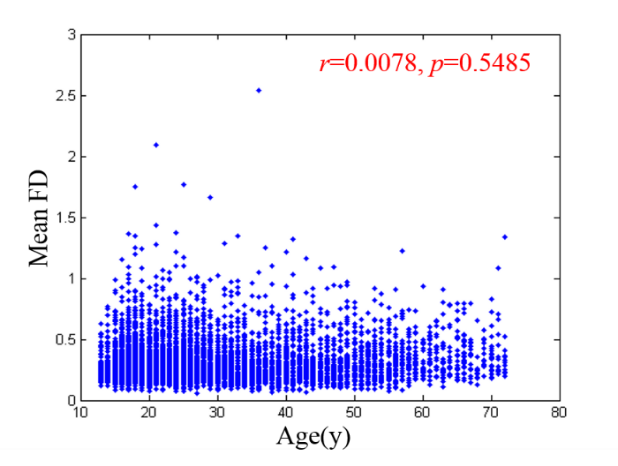


**Figure S5.** The correlation between mean FD and age for functional data.


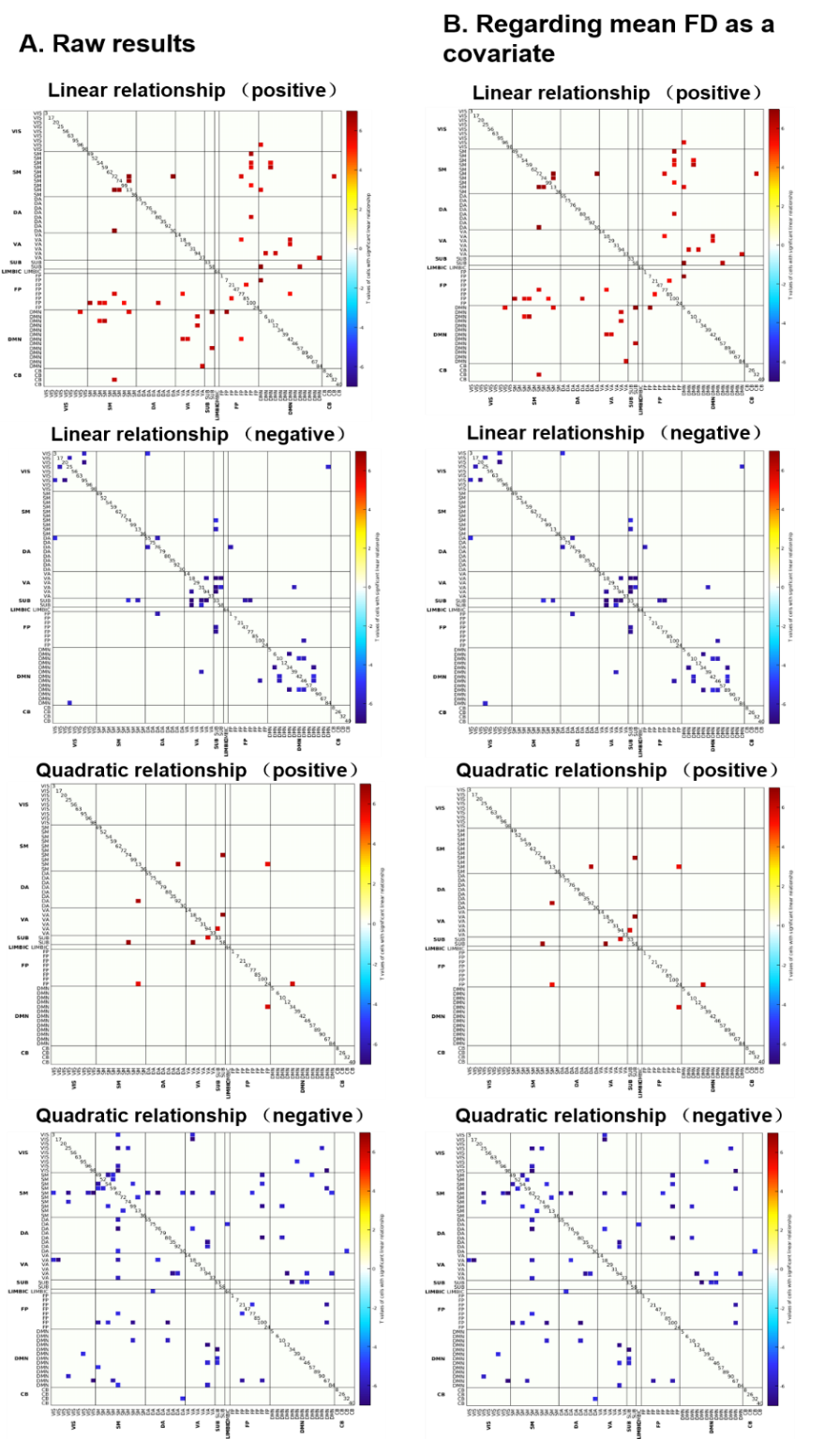


**Figure S6.** The comparison of relationship between FNC and age before (A) and after (B) regarding mean FD as a covariate.


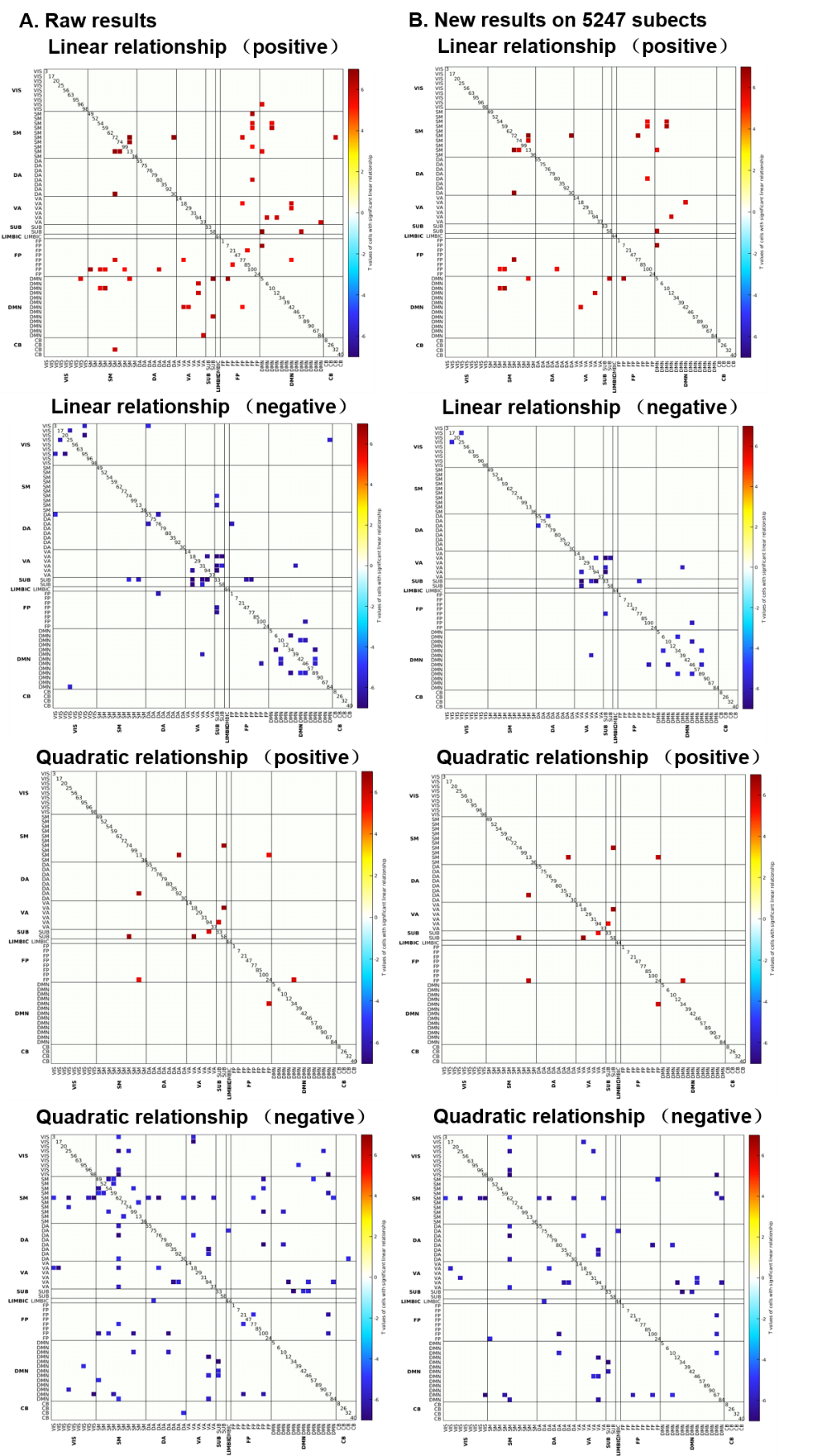


**Figure S7.** The comparison of relationship between FNC and age before (A) and after (B) removing 720 scans with mean FD larger than 0.5.
